# Ocular Glymphatic Dysfunction as a Potential Link Between Obstructive Sleep Apnea and Retinal Structural Changes. Comment on Pusic Sesar et al. Multimodal Assessment of Ocular Parameters in Patients with Severe Obstructive Sleep Apnea with Emphasis on Retinal Structural Changes. *Life* 2025, *15*, 1307

**DOI:** 10.3390/life16020214

**Published:** 2026-01-28

**Authors:** Peter Wostyn, Maiken Nedergaard

**Affiliations:** 1 Department of Psychiatry, Psychiatric Center Sint-Amandus, 8730 Beernem, Belgium; 2 Center for Translational Neuromedicine, Faculty of Health and Medical Sciences, University of Copenhagen, 2200 Copenhagen, Denmark; 3 Center for Translational Neuromedicine, University of Rochester Medical Center, Rochester, NY 14642, USA

**Keywords:** amyloid-β, ganglion cell layer, glaucoma, glymphatic system, intracranial pressure, intraocular pressure, obstructive sleep apnea, ocular glymphatic clearance, retinal nerve fiber layer

We read the article by Pusic Sesar and colleagues [1] entitled “Multimodal Assessment of Ocular Parameters in Patients with Severe Obstructive Sleep Apnea with Emphasis on Retinal Structural Changes” with great interest. The authors should be congratulated for their valuable contribution, demonstrating significant thinning of the ganglion cell layer in patients with severe obstructive sleep apnea (OSA) compared with healthy controls, which may reflect early glaucomatous changes and a potential link between OSA and glaucoma. Furthermore, a trend toward a reduction in retinal nerve fiber layer (RNFL) thickness was observed in the OSA group, although this did not reach statistical significance. The authors suggested that this outcome could be partly explained by the small sample size and limited statistical power, which may mask subtle yet clinically relevant effects. In addition, they discussed several pathophysiological mechanisms that may contribute to glaucomatous damage in OSA, including hypoxia, vascular dysregulation, and elevated intraocular pressure (IOP). They observed slightly higher IOP values in OSA patients than in controls, though without statistical significance. In addition to these considerations, we propose that glymphatic mechanisms may provide a complementary, IOP-independent pathway contributing to glaucomatous changes in OSA.

The glymphatic system, first identified in 2012, is a brain-wide waste clearance pathway in which cerebrospinal fluid (CSF) enters the brain via periarterial spaces and exchanges with interstitial fluid (ISF) within the parenchyma, facilitating the removal of solutes, including amyloid-β (Aβ) [2]. The resulting CSF-ISF mixture is then cleared along perivenous pathways and drains into meningeal and cervical lymphatic vessels [2]. Impaired sleep has been associated with reduced glymphatic clearance of Aβ in the brain [3]. In addition, studies in both rodents and humans support the existence of retrograde glymphatic-like fluid transport from the brain to the optic nerve [4,5,6,7,8]. We recently proposed that poor sleep may similarly impair the clearance function of this retrograde ocular glymphatic pathway, leading to waste accumulation at the optic nerve head and thereby increasing susceptibility to glaucomatous damage, particularly in normal-tension glaucoma [9]. Supporting this hypothesis, a subsequent study demonstrated that short sleep duration (<6 h) was significantly associated with thinner RNFL compared with the reference category (6–8 h), and also highlighted a significant association between clinically diagnosed insomnia and sleep apnea syndrome and glaucoma incidence [10]. Notably, the link between short sleep duration and thinner RNFL, despite IOP being highest in the 7–8 h group, suggests that RNFL thinning in short sleepers cannot simply be attributed to pressure-related effects but may involve alternative pathways [10]. In patients with OSA, disrupted sleep may similarly increase Aβ-related risk through impaired glymphatic clearance [10].

Beyond sleep-related impairment of retrograde ocular glymphatic clearance in OSA, we would like to highlight an additional glymphatic-related mechanism involving disrupted anterograde ocular glymphatic transport. Wang and colleagues [7] recently identified an “anterograde ocular glymphatic clearance system” in rodents that removes Aβ and other metabolites from the intraocular space via retinal ganglion cell axons and perivenous spaces of the retina and optic nerve head. After crossing the lamina barrier, intra-axonal Aβ is cleared through perivenous spaces in the optic nerve and drained into lymphatic vessels. Efflux along this pathway is driven by the trans-lamina cribrosa pressure difference (the difference between IOP and intracranial pressure (ICP)), with higher ICP suppressing clearance [7]. Several clinical studies have demonstrated that obstructive sleep apnea is associated with transient elevations in ICP during apneic episodes [11]. During obstructive apneas, repeated inspiratory movements against a blocked airway generate markedly negative intrathoracic pleural pressure, which enhances venous return and initially lowers ICP [12]. As apnea persists, excessive venous return to the heart elevates central venous pressure (CVP), ultimately reducing cerebral venous outflow [12]. This leads to venous congestion and elevated ICP [12]. Once breathing resumes, the intrapleural pressure rapidly shifts to positive, further increasing both CVP and ICP [12]. This rise in ICP may reduce the trans-lamina cribrosa pressure difference, thereby impeding intra-axonal efflux across the lamina cribrosa, depending on concurrent changes in IOP. Furthermore, impaired cerebral venous outflow may lead to venous distension and narrowing of perivenous spaces, reducing glymphatic clearance of Aβ and other metabolites. In the optic nerve, venous congestion may similarly compromise perivenous pathways, further impairing ocular glymphatic clearance. We hypothesize that these glymphatic mechanisms could constitute one of several IOP-independent pathways through which OSA may contribute to early glaucomatous changes.

Further research is warranted to determine whether ocular glymphatic transport is disrupted in OSA, and to clarify how it may interact with other factors contributing to glaucomatous neurodegeneration. If confirmed, ocular glymphatic dysfunction may represent a critical IOP-independent mechanism linking OSA to glaucoma, with potential therapeutic implications.
